# 3,3,6,6,9,9-Hexamethyl-2,3,4,5,6,7,8,9-octa­hydro-1*H*-xanthene-1,8-dione

**DOI:** 10.1107/S1600536808042189

**Published:** 2009-01-08

**Authors:** Xiaofen Xie, Yuying Zhang, Jiefeng Liang, Yulin Zhu

**Affiliations:** a School of Chemistry and Environment, South China Normal University, Guangzhou 510006, People’s Republic of China

## Abstract

The title compound, C_19_H_26_O_3_, was synthesized directly from the condensation of 5,5-dimethyl­cyclo­hexane-1,3-dione with malononitrile catalysed by palladium chloride: there are two molecules in the asymmetric unit.

## Related literature

For previous reports of the title compound, see: Hirsjarvi (1946 ▶); Sellstedt (1972 ▶).
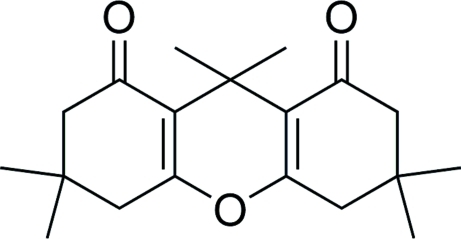


## Experimental

### 

#### Crystal data

C_19_H_26_O_3_
*M* *_r_* = 302.40
Monoclinic, 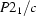

*a* = 12.1688 (19) Å
*b* = 11.7055 (18) Å
*c* = 24.365 (4) Å
β = 103.595 (2)°
*V* = 3373.4 (9) Å^3^
*Z* = 8
Mo *K*α radiation
μ = 0.08 mm^−1^
*T* = 298 (2) K
0.30 × 0.20 × 0.15 mm

#### Data collection

Bruker APEXII area-detector diffractometer
Absorption correction: multi-scan (*SADABS*; Sheldrick, 2004 ▶) *T*_min_ = 0.970, *T*_max_ = 0.981
20408 measured reflections
7856 independent reflections
4312 reflections with *I* > 2σ(*I*)
*R*_int_ = 0.042

#### Refinement

*R*[*F* ^2^ > 2σ(*F* ^2^)] = 0.058
*wR*(*F* ^2^) = 0.165
*S* = 1.05
7856 reflections
410 parameters
H-atom parameters constrained
Δρ_max_ = 0.22 e Å^−3^
Δρ_min_ = −0.16 e Å^−3^

### 

Data collection: *APEX2* (Bruker, 2004 ▶); cell refinement: *SAINT* (Bruker, 2004 ▶); data reduction: *SAINT*; program(s) used to solve structure: *SHELXS97* (Sheldrick, 2008 ▶); program(s) used to refine structure: *SHELXL97* (Sheldrick, 2008 ▶); molecular graphics: *XP* in *SHELXTL* (Sheldrick, 2008 ▶); software used to prepare material for publication: *SHELXTL*.

## Supplementary Material

Crystal structure: contains datablocks global, I. DOI: 10.1107/S1600536808042189/zl2156sup1.cif

Structure factors: contains datablocks I. DOI: 10.1107/S1600536808042189/zl2156Isup2.hkl

Additional supplementary materials:  crystallographic information; 3D view; checkCIF report

## Figures and Tables

**Fig. 1.**
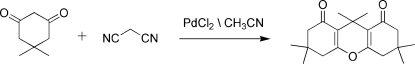
Palladium-catalyzed Synthesis of 3,4,5,6,7,9-hexahydro-3,3,6,6,9,9-hexamethyl-1H-xanthene-1,8(2H)-dione.

**Fig. 2.**
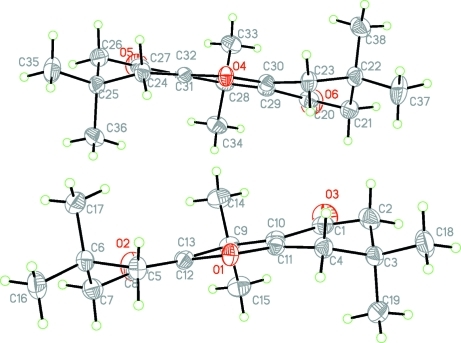
View of the title compound showing the atom-labelling scheme. Ellipsoids are drawn at the 50% probability level.

## supplementary crystallographic information

### Comment

The title compound, 3,4,5,6,7,9-hexahydro-3,3,6,6,9,9-hexamethyl-1*H*-xanthene-1,8(2*H*)-dione, was reported in 1946 (Hirsjarvi,1946; Sellstedt,1972). In our experiment, The reaction between 5,5-dimethyl-1,3-cyclohexanedione with malononitrile afforded 3,4,5,6,7,9-hexahydro-3,3,6,6,9,9-hexamethyl-1*H*-xanthene-1,8(2*H*)-dione in excellent yield in the presence of palladium chloride at 353 K for 5 h. (Fig. 1). The single crystals of the compound were obtained from ethanol as colourless block-shaped and crystallized in the space group P 21/c. There are no unusual bond lengths and angles in the compound. The O1—C11—C10—C1 torsion angle of 171.8 together with the O1—C12—C13—C8 torsion angle of 173.1 comfirms the bonds around the O1 atom are not coplanar. The O2—C8—C13—C12 torsion angle of 174.5 together with normal O2=C8 and C12=C13 bond lengths and the O3—C1—C10—C11 torsion angle of 177.0 together with normal O3=C1 and C10=C11 bond lengths indicate the presence of conjugation between these two double bonds. The C7—C8—C13—C12 torsion angle of -6.7 (3) together with the C5—C12—C13—C8 torsion angle of -5.9 (3) and the C2—C1—C10—C11 torsion angle of -0.3 (3) together with the C1—C10—C11—C4 torsion angle of -8.0 (3) exhibited the two rings are in a half-chair or envelope conformation. The C16 and C17 methyls were on the opposite to the C18 and C19 methyls, together with the C35 and C36 methyls were on the opposite to the C37 and C38 methyls,these confirmed the molecules conformation were *trans*. X-ray single-crystal diffraction reveals that there are crystallographically two independent mirror-image structures in the asymmetric unit.

### Experimental

A mixture of 5,5-dimethyl-1,3-cyclohexanedione (10 mmol), malononitrile (10 mmol), and palladium chloride (0.01 mmol) was refluxed in acetonitrile(12 ml) under 353 K for 5 h. After being cooled to room temperature, the reaction mixture was poured into water. The residue was filtered through a silica pad, washed twice with water, and then dried under vacuum to yield the product in yield 92%. The crystalloid product was dissolved in ethanol. Colourless block-shaped single crystals suitable for X-ray structure analysis were obtained by slow evaporation from ethanol at room temperature.

### Refinement

The H atoms were positioned geometrically and allowed to ride on their parent atoms, with C—H = 0.93–0.97 Å, and *U*_iso_ = 1.2–1.5 *U*_eq_(parent atom).

### Crystal data

**Table:**

C_19_H_26_O_3_	*F*(000) = 1312.0
*M**_r_* = 302.40	*D*_x_ = 1.191 Mg m^−^^3^
Monoclinic, *P*2_1_/*c*	Mo *K*α radiation, λ = 0.71073 Å
Hall symbol: -P 2ybc	Cell parameters from 3174 reflections
*a* = 12.1688 (19) Å	θ = 2.5–21.9°
*b* = 11.7055 (18) Å	µ = 0.08 mm^−^^1^
*c* = 24.365 (4) Å	*T* = 298 K
β = 103.595 (2)°	Block, colourless
*V* = 3373.4 (9) Å^3^	0.30 × 0.20 × 0.15 mm
*Z* = 8	

### Data collection

**Table:**

Bruker APEXII area-detector diffractometer	7856 independent reflections
Radiation source: fine-focus sealed tube	4312 reflections with *I* > 2σ(*I*)
graphite	*R*_int_ = 0.042
φ and ω scans	θ_max_ = 27.9°, θ_min_ = 1.7°
Absorption correction: multi-scan (*SADABS*; Sheldrick, 2004)	*h* = −15→16
*T*_min_ = 0.970, *T*_max_ = 0.981	*k* = −15→15
20408 measured reflections	*l* = −28→32

### Refinement

**Table:**

Refinement on *F*^2^	Secondary atom site location: difference Fourier map
Least-squares matrix: full	Hydrogen site location: inferred from neighbouring sites
*R*[*F*^2^ > 2σ(*F*^2^)] = 0.058	H-atom parameters constrained
*wR*(*F*^2^) = 0.165	*w* = 1/[σ^2^(*F*_o_^2^) + (0.0568*P*)^2^ + 0.7487*P*] where *P* = (*F*_o_^2^ + 2*F*_c_^2^)/3
*S* = 1.05	(Δ/σ)_max_ < 0.001
7856 reflections	Δρ_max_ = 0.22 e Å^−^^3^
410 parameters	Δρ_min_ = −0.16 e Å^−^^3^
0 restraints	Extinction correction: *SHELXL97* (Sheldrick, 2008), Fc^*^=kFc[1+0.001xFc^2^λ^3^/sin(2θ)]^-1/4^
Primary atom site location: structure-invariant direct methods	Extinction coefficient: 0.0012 (4)

### Special details

**Table:**

Geometry. All e.s.d.'s (except the e.s.d. in the dihedral angle between two l.s. planes) are estimated using the full covariance matrix. The cell e.s.d.'s are taken into account individually in the estimation of e.s.d.'s in distances, angles and torsion angles; correlations between e.s.d.'s in cell parameters are only used when they are defined by crystal symmetry. An approximate (isotropic) treatment of cell e.s.d.'s is used for estimating e.s.d.'s involving l.s. planes.
Refinement. Refinement of *F*^2^ against ALL reflections. The weighted *R*-factor *wR* and goodness of fit *S* are based on *F*^2^, conventional *R*-factors *R* are based on *F*, with *F* set to zero for negative *F*^2^. The threshold expression of *F*^2^ > σ(*F*^2^) is used only for calculating *R*-factors(gt) *etc*. and is not relevant to the choice of reflections for refinement. *R*-factors based on *F*^2^ are statistically about twice as large as those based on *F*, and *R*- factors based on ALL data will be even larger.

### Fractional atomic coordinates and isotropic or equivalent isotropic displacement parameters (Å^2^)

**Table:**

	*x*	*y*	*z*	*U*_iso_*/*U*_eq_	
C1	0.46830 (19)	0.7292 (2)	0.14266 (11)	0.0522 (6)	
C2	0.4840 (2)	0.8027 (2)	0.09431 (11)	0.0595 (7)	
H2A	0.4396	0.8718	0.0935	0.071*	
H2B	0.4545	0.7621	0.0593	0.071*	
C3	0.60593 (19)	0.83619 (19)	0.09702 (9)	0.0467 (6)	
C4	0.64836 (19)	0.89530 (18)	0.15383 (9)	0.0435 (5)	
H4A	0.7297	0.9045	0.1609	0.052*	
H4B	0.6151	0.9709	0.1521	0.052*	
C5	0.76147 (19)	0.8708 (2)	0.34933 (9)	0.0458 (5)	
H5A	0.7522	0.9529	0.3454	0.055*	
H5B	0.8388	0.8527	0.3483	0.055*	
C6	0.74147 (19)	0.8344 (2)	0.40666 (9)	0.0457 (5)	
C7	0.7276 (2)	0.7050 (2)	0.40465 (10)	0.0546 (6)	
H7A	0.7977	0.6705	0.4006	0.066*	
H7B	0.7135	0.6790	0.4402	0.066*	
C8	0.6324 (2)	0.6646 (2)	0.35692 (11)	0.0514 (6)	
C9	0.53208 (17)	0.67934 (18)	0.25027 (10)	0.0428 (5)	
C10	0.54395 (17)	0.74911 (18)	0.19899 (9)	0.0408 (5)	
C11	0.62087 (17)	0.83152 (17)	0.20159 (9)	0.0369 (5)	
C12	0.68309 (17)	0.81411 (18)	0.30053 (9)	0.0379 (5)	
C13	0.61809 (17)	0.72250 (17)	0.30206 (9)	0.0404 (5)	
C14	0.41224 (19)	0.6936 (2)	0.26027 (12)	0.0615 (7)	
H14A	0.3957	0.7734	0.2626	0.092*	
H14B	0.4084	0.6566	0.2949	0.092*	
H14C	0.3580	0.6597	0.2295	0.092*	
C15	0.5559 (2)	0.55184 (19)	0.23925 (12)	0.0607 (7)	
H15A	0.5040	0.5265	0.2054	0.091*	
H15B	0.5463	0.5063	0.2706	0.091*	
H15C	0.6320	0.5440	0.2350	0.091*	
C16	0.8430 (2)	0.8683 (2)	0.45361 (10)	0.0656 (7)	
H16A	0.8306	0.8446	0.4894	0.098*	
H16B	0.8525	0.9497	0.4536	0.098*	
H16C	0.9098	0.8320	0.4474	0.098*	
C17	0.6351 (2)	0.8909 (2)	0.41695 (10)	0.0584 (7)	
H17A	0.5718	0.8723	0.3865	0.088*	
H17B	0.6453	0.9723	0.4189	0.088*	
H17C	0.6213	0.8637	0.4519	0.088*	
C18	0.6127 (2)	0.9184 (2)	0.04973 (10)	0.0707 (8)	
H18A	0.5837	0.8820	0.0140	0.106*	
H18B	0.6900	0.9400	0.0529	0.106*	
H18C	0.5686	0.9853	0.0525	0.106*	
C19	0.6760 (2)	0.7294 (2)	0.09236 (11)	0.0636 (7)	
H19A	0.6739	0.6784	0.1229	0.095*	
H19B	0.7528	0.7512	0.0941	0.095*	
H19C	0.6452	0.6918	0.0571	0.095*	
C20	0.0368 (2)	0.67067 (19)	0.14075 (11)	0.0510 (6)	
C21	0.0876 (2)	0.7132 (2)	0.09394 (10)	0.0540 (6)	
H21A	0.0415	0.6872	0.0581	0.065*	
H21B	0.1624	0.6804	0.0984	0.065*	
C22	0.0969 (2)	0.84297 (19)	0.09278 (9)	0.0479 (6)	
C23	0.16921 (19)	0.87919 (19)	0.15058 (9)	0.0465 (6)	
H23A	0.2480	0.8635	0.1518	0.056*	
H23B	0.1614	0.9609	0.1550	0.056*	
C24	0.23595 (19)	0.89884 (18)	0.34621 (9)	0.0430 (5)	
H24A	0.2042	0.9745	0.3475	0.052*	
H24B	0.3116	0.9077	0.3404	0.052*	
C25	0.24293 (19)	0.83916 (18)	0.40265 (9)	0.0446 (5)	
C26	0.1227 (2)	0.8058 (2)	0.40333 (10)	0.0545 (6)	
H26A	0.1236	0.7665	0.4385	0.065*	
H26B	0.0782	0.8748	0.4026	0.065*	
C27	0.06587 (19)	0.7305 (2)	0.35496 (10)	0.0488 (6)	
C28	0.03382 (17)	0.68151 (17)	0.24750 (10)	0.0422 (5)	
C29	0.07195 (17)	0.72731 (17)	0.19632 (9)	0.0406 (5)	
C30	0.13723 (17)	0.81928 (17)	0.19853 (9)	0.0383 (5)	
C31	0.16534 (17)	0.83464 (17)	0.29759 (9)	0.0360 (5)	
C32	0.09154 (17)	0.75143 (17)	0.29926 (9)	0.0394 (5)	
C33	−0.09545 (18)	0.6935 (2)	0.23688 (11)	0.0588 (7)	
H33A	−0.1209	0.6584	0.2673	0.088*	
H33B	−0.1306	0.6566	0.2020	0.088*	
H33C	−0.1154	0.7730	0.2347	0.088*	
C34	0.0699 (2)	0.55488 (18)	0.25848 (11)	0.0587 (7)	
H34A	0.1502	0.5485	0.2630	0.088*	
H34B	0.0320	0.5090	0.2270	0.088*	
H34C	0.0499	0.5287	0.2922	0.088*	
C35	0.2903 (3)	0.9214 (2)	0.45098 (10)	0.0679 (8)	
H35A	0.2427	0.9877	0.4476	0.102*	
H35B	0.3653	0.9442	0.4493	0.102*	
H35C	0.2928	0.8842	0.4864	0.102*	
C36	0.3184 (2)	0.7331 (2)	0.40887 (11)	0.0617 (7)	
H36A	0.3189	0.6961	0.4441	0.093*	
H36B	0.3939	0.7554	0.4081	0.093*	
H36C	0.2895	0.6814	0.3783	0.093*	
C37	0.1548 (3)	0.8809 (3)	0.04655 (10)	0.0747 (9)	
H37A	0.2246	0.8400	0.0504	0.112*	
H37B	0.1700	0.9614	0.0500	0.112*	
H37C	0.1063	0.8651	0.0102	0.112*	
C38	−0.0209 (2)	0.8958 (2)	0.08221 (11)	0.0671 (8)	
H38A	−0.0663	0.8680	0.0471	0.101*	
H38B	−0.0150	0.9775	0.0805	0.101*	
H38C	−0.0555	0.8753	0.1124	0.101*	
O1	0.68792 (12)	0.86994 (12)	0.25180 (6)	0.0404 (4)	
O2	0.57242 (17)	0.58530 (16)	0.36467 (8)	0.0799 (6)	
O3	0.39140 (15)	0.65934 (16)	0.13512 (8)	0.0762 (6)	
O4	0.18654 (12)	0.87463 (11)	0.24804 (6)	0.0397 (3)	
O5	−0.00287 (15)	0.65889 (16)	0.36175 (8)	0.0731 (6)	
O6	−0.02878 (17)	0.58965 (16)	0.13253 (8)	0.0789 (6)	

### Atomic displacement parameters (Å^2^)

**Table:**

	*U*^11^	*U*^22^	*U*^33^	*U*^12^	*U*^13^	*U*^23^
C1	0.0433 (13)	0.0515 (14)	0.0581 (16)	0.0010 (11)	0.0050 (12)	−0.0166 (12)
C2	0.0574 (16)	0.0614 (16)	0.0499 (15)	0.0057 (12)	−0.0073 (12)	−0.0104 (13)
C3	0.0528 (14)	0.0484 (13)	0.0344 (12)	0.0088 (11)	0.0015 (10)	−0.0056 (10)
C4	0.0479 (13)	0.0440 (13)	0.0369 (12)	−0.0008 (10)	0.0066 (10)	−0.0031 (10)
C5	0.0502 (13)	0.0510 (13)	0.0354 (12)	−0.0079 (11)	0.0082 (10)	−0.0010 (10)
C6	0.0504 (13)	0.0521 (14)	0.0348 (12)	0.0028 (11)	0.0104 (10)	0.0053 (10)
C7	0.0612 (15)	0.0549 (15)	0.0500 (15)	0.0085 (12)	0.0176 (12)	0.0129 (12)
C8	0.0574 (15)	0.0407 (13)	0.0609 (16)	0.0021 (11)	0.0235 (13)	0.0060 (12)
C9	0.0393 (12)	0.0371 (11)	0.0535 (14)	−0.0044 (9)	0.0140 (11)	−0.0071 (10)
C10	0.0368 (11)	0.0378 (12)	0.0467 (14)	−0.0008 (9)	0.0078 (10)	−0.0111 (10)
C11	0.0353 (11)	0.0389 (11)	0.0351 (12)	0.0021 (9)	0.0053 (9)	−0.0082 (9)
C12	0.0418 (12)	0.0390 (12)	0.0350 (12)	0.0011 (9)	0.0136 (9)	−0.0007 (9)
C13	0.0422 (12)	0.0349 (11)	0.0468 (14)	0.0015 (9)	0.0157 (10)	0.0002 (10)
C14	0.0454 (14)	0.0652 (17)	0.0780 (19)	−0.0044 (12)	0.0229 (13)	−0.0024 (14)
C15	0.0700 (17)	0.0360 (13)	0.0767 (19)	−0.0017 (12)	0.0187 (14)	−0.0094 (13)
C16	0.0697 (18)	0.086 (2)	0.0382 (14)	−0.0047 (15)	0.0060 (13)	0.0042 (14)
C17	0.0675 (17)	0.0632 (17)	0.0479 (15)	0.0094 (13)	0.0202 (13)	−0.0003 (12)
C18	0.090 (2)	0.080 (2)	0.0361 (15)	0.0091 (16)	0.0020 (14)	0.0058 (14)
C19	0.0747 (18)	0.0656 (17)	0.0505 (16)	0.0179 (14)	0.0149 (14)	−0.0094 (13)
C20	0.0508 (14)	0.0369 (12)	0.0601 (16)	−0.0020 (11)	0.0028 (12)	−0.0080 (11)
C21	0.0586 (15)	0.0504 (14)	0.0481 (15)	0.0023 (12)	0.0026 (12)	−0.0144 (12)
C22	0.0593 (15)	0.0455 (13)	0.0347 (13)	−0.0023 (11)	0.0028 (11)	−0.0064 (10)
C23	0.0540 (14)	0.0470 (13)	0.0367 (13)	−0.0097 (11)	0.0071 (11)	−0.0031 (10)
C24	0.0509 (13)	0.0408 (12)	0.0376 (12)	−0.0035 (10)	0.0108 (10)	0.0011 (10)
C25	0.0578 (14)	0.0406 (12)	0.0355 (12)	0.0040 (10)	0.0110 (11)	0.0043 (10)
C26	0.0688 (16)	0.0521 (15)	0.0492 (15)	0.0086 (12)	0.0272 (13)	0.0120 (12)
C27	0.0480 (13)	0.0439 (13)	0.0578 (16)	0.0043 (11)	0.0189 (12)	0.0135 (12)
C28	0.0379 (12)	0.0334 (11)	0.0535 (14)	−0.0015 (9)	0.0073 (10)	0.0046 (10)
C29	0.0368 (11)	0.0338 (11)	0.0483 (14)	0.0000 (9)	0.0043 (10)	−0.0015 (10)
C30	0.0384 (11)	0.0366 (11)	0.0377 (12)	−0.0017 (9)	0.0044 (9)	−0.0024 (10)
C31	0.0394 (11)	0.0342 (11)	0.0353 (12)	0.0035 (9)	0.0106 (9)	0.0040 (9)
C32	0.0361 (11)	0.0345 (11)	0.0484 (14)	0.0035 (9)	0.0115 (10)	0.0072 (10)
C33	0.0401 (13)	0.0623 (16)	0.0722 (18)	−0.0040 (11)	0.0093 (12)	0.0045 (14)
C34	0.0663 (16)	0.0340 (12)	0.0734 (18)	0.0012 (11)	0.0113 (14)	0.0067 (12)
C35	0.102 (2)	0.0620 (17)	0.0390 (15)	−0.0051 (15)	0.0151 (14)	−0.0038 (13)
C36	0.0672 (17)	0.0568 (16)	0.0556 (17)	0.0152 (13)	0.0033 (13)	0.0064 (13)
C37	0.106 (2)	0.080 (2)	0.0388 (15)	−0.0223 (17)	0.0170 (15)	−0.0121 (14)
C38	0.0777 (19)	0.0567 (16)	0.0566 (17)	0.0112 (14)	−0.0052 (14)	−0.0057 (13)
O1	0.0464 (8)	0.0427 (8)	0.0314 (8)	−0.0093 (7)	0.0077 (7)	−0.0036 (7)
O2	0.0988 (15)	0.0631 (12)	0.0806 (14)	−0.0254 (11)	0.0266 (12)	0.0180 (10)
O3	0.0629 (12)	0.0746 (13)	0.0823 (14)	−0.0251 (10)	−0.0007 (10)	−0.0200 (11)
O4	0.0473 (8)	0.0386 (8)	0.0323 (8)	−0.0099 (6)	0.0074 (7)	−0.0015 (7)
O5	0.0756 (13)	0.0690 (12)	0.0833 (14)	−0.0177 (10)	0.0358 (11)	0.0164 (10)
O6	0.0907 (14)	0.0615 (12)	0.0799 (14)	−0.0332 (11)	0.0106 (11)	−0.0219 (10)

### Geometric parameters (Å, °)

**Table:**

C1—O3	1.224 (3)	C20—O6	1.226 (3)
C1—C10	1.480 (3)	C20—C29	1.478 (3)
C1—C2	1.507 (4)	C20—C21	1.504 (3)
C2—C3	1.521 (3)	C21—C22	1.524 (3)
C2—H2A	0.9700	C21—H21A	0.9700
C2—H2B	0.9700	C21—H21B	0.9700
C3—C18	1.518 (3)	C22—C37	1.528 (3)
C3—C4	1.525 (3)	C22—C38	1.527 (3)
C3—C19	1.532 (3)	C22—C23	1.535 (3)
C4—C11	1.485 (3)	C23—C30	1.491 (3)
C4—H4A	0.9700	C23—H23A	0.9700
C4—H4B	0.9700	C23—H23B	0.9700
C5—C12	1.493 (3)	C24—C31	1.493 (3)
C5—C6	1.533 (3)	C24—C25	1.527 (3)
C5—H5A	0.9700	C24—H24A	0.9700
C5—H5B	0.9700	C24—H24B	0.9700
C6—C7	1.524 (3)	C25—C26	1.518 (3)
C6—C17	1.526 (3)	C25—C35	1.526 (3)
C6—C16	1.526 (3)	C25—C36	1.530 (3)
C7—C8	1.511 (3)	C26—C27	1.504 (3)
C7—H7A	0.9700	C26—H26A	0.9700
C7—H7B	0.9700	C26—H26B	0.9700
C8—O2	1.223 (3)	C27—O5	1.223 (3)
C8—C13	1.472 (3)	C27—C32	1.483 (3)
C9—C13	1.524 (3)	C28—C29	1.526 (3)
C9—C10	1.528 (3)	C28—C32	1.529 (3)
C9—C14	1.543 (3)	C28—C33	1.539 (3)
C9—C15	1.556 (3)	C28—C34	1.551 (3)
C10—C11	1.335 (3)	C29—C30	1.332 (3)
C11—O1	1.377 (2)	C30—O4	1.376 (2)
C12—C13	1.338 (3)	C31—C32	1.332 (3)
C12—O1	1.369 (2)	C31—O4	1.375 (2)
C14—H14A	0.9600	C33—H33A	0.9600
C14—H14B	0.9600	C33—H33B	0.9600
C14—H14C	0.9600	C33—H33C	0.9600
C15—H15A	0.9600	C34—H34A	0.9600
C15—H15B	0.9600	C34—H34B	0.9600
C15—H15C	0.9600	C34—H34C	0.9600
C16—H16A	0.9600	C35—H35A	0.9600
C16—H16B	0.9600	C35—H35B	0.9600
C16—H16C	0.9600	C35—H35C	0.9600
C17—H17A	0.9600	C36—H36A	0.9600
C17—H17B	0.9600	C36—H36B	0.9600
C17—H17C	0.9600	C36—H36C	0.9600
C18—H18A	0.9600	C37—H37A	0.9600
C18—H18B	0.9600	C37—H37B	0.9600
C18—H18C	0.9600	C37—H37C	0.9600
C19—H19A	0.9600	C38—H38A	0.9600
C19—H19B	0.9600	C38—H38B	0.9600
C19—H19C	0.9600	C38—H38C	0.9600
			
O3—C1—C10	122.1 (2)	O6—C20—C21	120.0 (2)
O3—C1—C2	119.8 (2)	C29—C20—C21	117.68 (19)
C10—C1—C2	118.0 (2)	C20—C21—C22	112.91 (19)
C1—C2—C3	114.49 (19)	C20—C21—H21A	109.0
C1—C2—H2A	108.6	C22—C21—H21A	109.0
C3—C2—H2A	108.6	C20—C21—H21B	109.0
C1—C2—H2B	108.6	C22—C21—H21B	109.0
C3—C2—H2B	108.6	H21A—C21—H21B	107.8
H2A—C2—H2B	107.6	C21—C22—C37	110.6 (2)
C18—C3—C4	109.5 (2)	C21—C22—C38	109.7 (2)
C18—C3—C2	110.9 (2)	C37—C22—C38	109.6 (2)
C4—C3—C2	105.95 (19)	C21—C22—C23	106.69 (18)
C18—C3—C19	109.3 (2)	C37—C22—C23	109.4 (2)
C4—C3—C19	111.21 (19)	C38—C22—C23	110.8 (2)
C2—C3—C19	110.0 (2)	C30—C23—C22	112.98 (18)
C11—C4—C3	113.11 (18)	C30—C23—H23A	109.0
C11—C4—H4A	109.0	C22—C23—H23A	109.0
C3—C4—H4A	109.0	C30—C23—H23B	109.0
C11—C4—H4B	109.0	C22—C23—H23B	109.0
C3—C4—H4B	109.0	H23A—C23—H23B	107.8
H4A—C4—H4B	107.8	C31—C24—C25	112.59 (18)
C12—C5—C6	113.17 (18)	C31—C24—H24A	109.1
C12—C5—H5A	108.9	C25—C24—H24A	109.1
C6—C5—H5A	108.9	C31—C24—H24B	109.1
C12—C5—H5B	108.9	C25—C24—H24B	109.1
C6—C5—H5B	108.9	H24A—C24—H24B	107.8
H5A—C5—H5B	107.8	C26—C25—C35	110.2 (2)
C7—C6—C17	110.1 (2)	C26—C25—C24	106.12 (18)
C7—C6—C5	106.66 (19)	C35—C25—C24	109.70 (19)
C17—C6—C5	110.60 (19)	C26—C25—C36	110.35 (19)
C7—C6—C16	110.3 (2)	C35—C25—C36	109.1 (2)
C17—C6—C16	109.4 (2)	C24—C25—C36	111.43 (19)
C5—C6—C16	109.68 (19)	C27—C26—C25	114.16 (19)
C8—C7—C6	113.27 (19)	C27—C26—H26A	108.7
C8—C7—H7A	108.9	C25—C26—H26A	108.7
C6—C7—H7A	108.9	C27—C26—H26B	108.7
C8—C7—H7B	108.9	C25—C26—H26B	108.7
C6—C7—H7B	108.9	H26A—C26—H26B	107.6
H7A—C7—H7B	107.7	O5—C27—C32	122.4 (2)
O2—C8—C13	122.8 (2)	O5—C27—C26	120.0 (2)
O2—C8—C7	119.8 (2)	C32—C27—C26	117.6 (2)
C13—C8—C7	117.4 (2)	C29—C28—C32	108.48 (17)
C13—C9—C10	108.75 (17)	C29—C28—C33	108.99 (18)
C13—C9—C14	108.91 (19)	C32—C28—C33	110.06 (18)
C10—C9—C14	110.23 (19)	C29—C28—C34	110.38 (19)
C13—C9—C15	109.97 (19)	C32—C28—C34	108.23 (18)
C10—C9—C15	108.33 (19)	C33—C28—C34	110.68 (18)
C14—C9—C15	110.63 (18)	C30—C29—C20	116.2 (2)
C11—C10—C1	115.7 (2)	C30—C29—C28	123.0 (2)
C11—C10—C9	123.2 (2)	C20—C29—C28	120.87 (18)
C1—C10—C9	121.10 (19)	C29—C30—O4	123.3 (2)
C10—C11—O1	122.7 (2)	C29—C30—C23	127.7 (2)
C10—C11—C4	127.6 (2)	O4—C30—C23	108.98 (17)
O1—C11—C4	109.66 (17)	C32—C31—O4	122.88 (19)
C13—C12—O1	123.57 (19)	C32—C31—C24	127.6 (2)
C13—C12—C5	127.2 (2)	O4—C31—C24	109.50 (17)
O1—C12—C5	109.20 (17)	C31—C32—C27	115.9 (2)
C12—C13—C8	116.7 (2)	C31—C32—C28	123.3 (2)
C12—C13—C9	122.6 (2)	C27—C32—C28	120.78 (18)
C8—C13—C9	120.74 (19)	C28—C33—H33A	109.5
C9—C14—H14A	109.5	C28—C33—H33B	109.5
C9—C14—H14B	109.5	H33A—C33—H33B	109.5
H14A—C14—H14B	109.5	C28—C33—H33C	109.5
C9—C14—H14C	109.5	H33A—C33—H33C	109.5
H14A—C14—H14C	109.5	H33B—C33—H33C	109.5
H14B—C14—H14C	109.5	C28—C34—H34A	109.5
C9—C15—H15A	109.5	C28—C34—H34B	109.5
C9—C15—H15B	109.5	H34A—C34—H34B	109.5
H15A—C15—H15B	109.5	C28—C34—H34C	109.5
C9—C15—H15C	109.5	H34A—C34—H34C	109.5
H15A—C15—H15C	109.5	H34B—C34—H34C	109.5
H15B—C15—H15C	109.5	C25—C35—H35A	109.5
C6—C16—H16A	109.5	C25—C35—H35B	109.5
C6—C16—H16B	109.5	H35A—C35—H35B	109.5
H16A—C16—H16B	109.5	C25—C35—H35C	109.5
C6—C16—H16C	109.5	H35A—C35—H35C	109.5
H16A—C16—H16C	109.5	H35B—C35—H35C	109.5
H16B—C16—H16C	109.5	C25—C36—H36A	109.5
C6—C17—H17A	109.5	C25—C36—H36B	109.5
C6—C17—H17B	109.5	H36A—C36—H36B	109.5
H17A—C17—H17B	109.5	C25—C36—H36C	109.5
C6—C17—H17C	109.5	H36A—C36—H36C	109.5
H17A—C17—H17C	109.5	H36B—C36—H36C	109.5
H17B—C17—H17C	109.5	C22—C37—H37A	109.5
C3—C18—H18A	109.5	C22—C37—H37B	109.5
C3—C18—H18B	109.5	H37A—C37—H37B	109.5
H18A—C18—H18B	109.5	C22—C37—H37C	109.5
C3—C18—H18C	109.5	H37A—C37—H37C	109.5
H18A—C18—H18C	109.5	H37B—C37—H37C	109.5
H18B—C18—H18C	109.5	C22—C38—H38A	109.5
C3—C19—H19A	109.5	C22—C38—H38B	109.5
C3—C19—H19B	109.5	H38A—C38—H38B	109.5
H19A—C19—H19B	109.5	C22—C38—H38C	109.5
C3—C19—H19C	109.5	H38A—C38—H38C	109.5
H19A—C19—H19C	109.5	H38B—C38—H38C	109.5
H19B—C19—H19C	109.5	C12—O1—C11	118.53 (16)
O6—C20—C29	122.3 (2)	C31—O4—C30	118.45 (16)
			
O3—C1—C2—C3	−148.9 (2)	C20—C21—C22—C23	58.0 (3)
C10—C1—C2—C3	34.3 (3)	C38—C22—C23—C30	74.9 (3)
C1—C2—C3—C18	−174.9 (2)	C21—C22—C23—C30	−44.4 (3)
C1—C2—C3—C4	−56.2 (3)	C37—C22—C23—C30	−164.1 (2)
C1—C2—C3—C19	64.1 (3)	C31—C24—C25—C26	−47.1 (2)
C18—C3—C4—C11	166.9 (2)	C31—C24—C25—C35	−166.0 (2)
C2—C3—C4—C11	47.2 (2)	C31—C24—C25—C36	73.1 (2)
C19—C3—C4—C11	−72.1 (3)	C35—C25—C26—C27	176.28 (19)
C12—C5—C6—C7	44.7 (3)	C24—C25—C26—C27	57.6 (2)
C12—C5—C6—C16	164.3 (2)	C36—C25—C26—C27	−63.2 (3)
C12—C5—C6—C17	−75.0 (3)	C25—C26—C27—O5	147.3 (2)
C16—C6—C7—C8	−176.7 (2)	C25—C26—C27—C32	−36.1 (3)
C17—C6—C7—C8	62.4 (3)	O6—C20—C29—C30	−175.4 (2)
C5—C6—C7—C8	−57.6 (2)	C21—C20—C29—C30	6.6 (3)
C6—C7—C8—O2	−140.9 (2)	O6—C20—C29—C28	4.2 (3)
C6—C7—C8—C13	40.3 (3)	C21—C20—C29—C28	−173.7 (2)
O3—C1—C10—C11	−177.1 (2)	C32—C28—C29—C30	−5.1 (3)
C2—C1—C10—C11	−0.3 (3)	C33—C28—C29—C30	114.7 (2)
O3—C1—C10—C9	2.4 (3)	C34—C28—C29—C30	−123.5 (2)
C2—C1—C10—C9	179.08 (19)	C32—C28—C29—C20	175.29 (18)
C13—C9—C10—C11	1.6 (3)	C33—C28—C29—C20	−64.9 (2)
C14—C9—C10—C11	120.9 (2)	C34—C28—C29—C20	56.8 (3)
C15—C9—C10—C11	−117.9 (2)	C20—C29—C30—O4	−173.07 (18)
C13—C9—C10—C1	−177.80 (18)	C28—C29—C30—O4	7.3 (3)
C14—C9—C10—C1	−58.5 (3)	C20—C29—C30—C23	7.0 (3)
C15—C9—C10—C1	62.7 (3)	C28—C29—C30—C23	−172.6 (2)
C1—C10—C11—O1	171.77 (18)	C22—C23—C30—C29	13.8 (3)
C9—C10—C11—O1	−7.6 (3)	C22—C23—C30—O4	−166.12 (18)
C1—C10—C11—C4	−8.0 (3)	C25—C24—C31—C32	16.4 (3)
C9—C10—C11—C4	172.54 (19)	C25—C24—C31—O4	−163.89 (17)
C3—C4—C11—C10	−17.6 (3)	O4—C31—C32—C27	−171.10 (18)
C3—C4—C11—O1	162.54 (17)	C24—C31—C32—C27	8.5 (3)
C6—C5—C12—C13	−15.0 (3)	O4—C31—C32—C28	8.0 (3)
C6—C5—C12—O1	165.85 (17)	C24—C31—C32—C28	−172.42 (19)
O1—C12—C13—C8	173.11 (18)	O5—C27—C32—C31	177.8 (2)
C5—C12—C13—C8	−5.9 (3)	C26—C27—C32—C31	1.3 (3)
O1—C12—C13—C9	−7.5 (3)	O5—C27—C32—C28	−1.2 (3)
C5—C12—C13—C9	173.5 (2)	C26—C27—C32—C28	−177.77 (19)
O2—C8—C13—C12	174.5 (2)	C29—C28—C32—C31	−2.3 (3)
C7—C8—C13—C12	−6.7 (3)	C33—C28—C32—C31	−121.5 (2)
O2—C8—C13—C9	−5.0 (3)	C34—C28—C32—C31	117.5 (2)
C7—C8—C13—C9	173.8 (2)	C29—C28—C32—C27	176.71 (18)
C10—C9—C13—C12	5.7 (3)	C33—C28—C32—C27	57.6 (2)
C14—C9—C13—C12	−114.5 (2)	C34—C28—C32—C27	−63.5 (2)
C15—C9—C13—C12	124.2 (2)	C13—C12—O1—C11	1.3 (3)
C10—C9—C13—C8	−174.88 (18)	C5—C12—O1—C11	−179.51 (17)
C14—C9—C13—C8	65.0 (2)	C10—C11—O1—C12	6.4 (3)
C15—C9—C13—C8	−56.4 (3)	C4—C11—O1—C12	−173.77 (17)
O6—C20—C21—C22	141.3 (2)	C32—C31—O4—C30	−6.1 (3)
C29—C20—C21—C22	−40.8 (3)	C24—C31—O4—C30	174.20 (17)
C20—C21—C22—C38	−62.1 (3)	C29—C30—O4—C31	−1.6 (3)
C20—C21—C22—C37	176.9 (2)	C23—C30—O4—C31	178.31 (17)
